# EGFR-induced suppression of HPV E6/E7 is mediated by microRNA-9-5p silencing of BRD4 protein in HPV-positive head and neck squamous cell carcinoma

**DOI:** 10.1038/s41419-022-05269-8

**Published:** 2022-11-04

**Authors:** Danupon Nantajit, Luana Presta, Thomas Sauter, Mahvash Tavassoli

**Affiliations:** 1 Centre for Host-Microbiome Interactions, King’s College London, London, United Kingdom; 2 Princess Srisavangavadhana College of Medicine, Chulabhorn Royal Academy, Bangkok, Thailand; 3 Department of Life Sciences and Medicine, University of Luxembourg, Belvaux, Luxembourg

**Keywords:** Head and neck cancer, Oncogenes, Preclinical research

## Abstract

EGFR upregulation is an established biomarker of treatment resistance and aggressiveness in head and neck cancers (HNSCC). EGFR-targeted therapies have shown benefits for HPV-negative HNSCC; surprisingly, inhibiting EGFR in HPV-associated HNSCC led to inferior therapeutic outcomes suggesting opposing roles for EGFR in the two HNSCC subtypes. The current study aimed to understand the link between EGFR and HPV-infected HNSCC particularly the regulation of HPV oncoproteins E6 and E7. We demonstrate that EGFR overexpression suppresses cellular proliferation and increases radiosensitivity of HPV-positive HNSCC cell lines. EGFR overexpression inhibited protein expression of BRD4, a known cellular transcriptional regulator of HPV E6/E7 expression and DNA damage repair facilitator. Inhibition of EGFR by cetuximab restored the expression of BRD4 leading to increased HPV E6 and E7 transcription. Concordantly, pharmacological inhibition of BRD4 led to suppression of HPV E6 and E7 transcription, delayed cellular proliferation and sensitised HPV-positive HNSCC cells to ionising radiation. This effect was shown to be mediated through EGFR-induced upregulation of microRNA-9-5p and consequent silencing of its target BRD4 at protein translational level, repressing HPV E6 and E7 transcription and restoring p53 tumour suppressor functions. These results suggest a novel mechanism for EGFR inhibition of HPV E6/E7 oncoprotein expression through an epigenetic pathway, independent of MAPK, but mediated through microRNA-9-5p/BRD4 regulation. Therefore, targeting EGFR may not be the best course of therapy for certain cancer types including HPV-positive HNSCC, while targeting specific signalling pathways such as BRD4 could provide a better and potentially new treatment to improve HNSCC therapeutic outcome.

## Introduction

Head and neck squamous cell carcinoma (HNSCC) is globally ranked the 6th most common malignancy with over 600,00 new cases annually [1]. The current treatment modalities for HNSCC include surgery, radiotherapy (RT) and chemotherapy (CT), often in combination [2]. These non-specific treatment modalities have severe adverse effects causing immense long-term suffering to the patients. The overall 5-year survival of patients with HNSCC is estimated to be 64% for oral cavity and pharynx cancers and 61% for larynx cancer [3]. HNSCC can be classified into two subtypes, HPV-negative subtype, mainly caused by smoking, and human papilloma virus (HPV) driven subtype. Despite the availability of HPV vaccines, the incidence of HPV-related HNSCC, specifically oropharyngeal HNSCC, is increasing notably in Northern Europe and North America [4].

Epidermal growth factor receptor (EGFR) is overexpressed in many tumour types including up to 90% of HNSCC and is linked to poorer prognosis, therapy resistance and locoregional failure [5]. Cetuximab, a monoclonal antibody against EGFR, has been the only approved targeted therapy for HNSCC since 2016, and was proposed to replace cisplatin to de-intensify current treatment regime for HPV-positive HNSCC to decrease adverse side effects [6–8]. Addition of cetuximab to RT was shown to be beneficial in both improvement in locoregional control and overall survival of HPV-negative HNSCC [9]. However, recent clinical trial results have questioned the benefit of cetuximab in combination with RT for HPV-positive oropharyngeal squamous cell carcinoma (OPSCC). Surprisingly, cetuximab showed significantly inferior outcome to RT combination with cisplatin [10]. Also, a pre-clinical study reports that EGFR inhibition failed to sensitise HPV-positive HNSCC to RT [11]. Additionally, we have recently reported that EGFR overexpression inhibits repair of radiation induced DNA damage and consequently radiosensitising HPV-positive HNSCC, in contrast to HPV-negative HNSCC [12]. Two other clinical studies have also shown poorer tumour control and patient survival rate in cetuximab-containing arms for HPV-positive patients [13, 14]. Collectively, these data warrant investigation into the roles of EGFR in different subtypes of HNSCC to help stratifying patients who are likely to benefit from EGFR-targeted therapy, particularly in HPV-positive tumours, in which inhibition of EGFR could potentially worsen therapy response.

It is well stablished that high-risk HPV oncoproteins E6 and E7 inhibit two main cell cycle regulators p53 and Rb, respectively, leading to genomic instability and tumorigenesis. However, the interplay between HPV and other cellular pathways such as receptor and intracellular signalling pathways remains elusive. There is some evidence for HPV oncoproteins E6 and E7 as well as E5 to alter cellular signalling pathways [15]. Both E6 and E7 oncoprotein are linked to enhanced epithelial-mesenchymal transition leading to tumour progression and metastasis [16]. Ectopic expression of HPV E6 in non-HPV cervical tumour cells enhances tumour aggressiveness as well as resistance to RT [17]. Activation of Sp1 via transcriptional activators MAPK/ERK has been defined as the mechanism of activating these oncogenic pathways [18]. Additionally, activation of transcription factor AP-1 through EGFR/MAPK/ERK can enhance E6/E7 transcription and drive the HPV oncogene expression [19].

One transcriptional regulator linked to HPV E6/E7 is BRD4 (Bromodomain-containing protein 4) [20]. As a member of the BET (bromodomain and extra-terminal domain) protein family, BRD4 binds to acetylated histones and transactivates a number of oncogenes as well as replication of papillomaviruses [21, 22]. BRD4 also has non-transcriptional roles relating to DNA damage repair, checkpoint activation and telomere maintenance [23]. BRD4 can form a superenhancer-like element and bind to HPV integration site to enhance E6/E7 transcription [24]. The protein often becomes dysregulated in cancer and, thus, has become a potential therapeutic target in several cancer types [25]. Transcriptional regulation of BRD4 is not often discussed; however, different microRNAs such as miR-29a and miR-200a have been described to regulate BRD4 at the epigenetic level [26, 27].

MicroRNA-9-5p (miR-9-5p) is a highly conserved microRNA across all vertebrate species. We have previously demonstrated that miR-9-5p acts as a growth suppressor in HNSCC by suppressing the expression of CXCR4 [28]. MiR-9-5p has also been shown to polarise macrophage into M1 phenotype and improve radiosensitivity of HPV-positive HNSCC [29]. Likewise, miR-9-5p inhibits NOX4 and suppresses TGF-β1-induced phenotypic transformation of fibroblasts in vitro and potentially leading to a better therapeutic response in HPV-positive HNSCC patients [30]. There is also evidence of miR-9-5p having an oncogenic activity; EGFR activation has been suggested to upregulate miR-9-5p in HPV-negative HNSCC and high miR-9-5p expression is associated with poor patient prognosis [31]. Regulation of BRD4 by the microRNA has been previously demonstrated in neonatal rat ventricular myocytes [32]. However, it is unclear whether in human cells, particularly in HNSCC, there is such microRNA-mediated regulation of BRD4. In this study, we investigated whether there is a link between EGFR and regulation of BRD4 through miR-9-5p and subsequently affecting HPV E6/E7 transcription influencing RT response in HPV-positive HNSCC cell lines.

## Materials and methods

### Antibodies and Reagents

Antibodies against EGFR, BRD4, p-ERK1/2, p53 and Rad51 were purchased from Cell Signaling Technology, catalogue numbers 4267, 13440, 9101, 48818 and 8875, respectively. Antibody against p63 was from Santa Cruz Biotechnology (sc-8431). P73 antibody was from Abcam (ab26123). Antibody against p16INK4a was purchased from ThermoFisher (MA5-17054). α-tubulin and β-actin antibodies were from Sigma, catalogue numbers T6074 and A5441, respectively. Selumetinib was purchased from Selleck (S1008). BRD4 inhibitor MZ1 was purchased from Abcam (ab230371).

### Cell culture and irradiation

HPV-positive SCC090, SCC152, SCC154 and HPV-negative SCC072 cells, from Professor Susanne Gollin, University of Pittsburgh (Pittsburgh, PA, USA), were cultured in MEM supplemented with 10% fetal bovine serum (FBS), 100 µg/ml gentamicin and 1× MEM non-essential amino acids and maintained in a humidified 5% CO2 incubator at 37 °C. Irradiation was achieved at indicated dose using gamma irradiator at room temperature. All experiments concerning cell treatments were achieved in the regular growth medium with 10% FBS.

### EGFR overexpression

SCC072 and SCC154 EGFR-overexpressing cells and their empty vector control (SCC072 vector and SCC154 vector) were previously established [12]. SCC090 and SCC152 EGFR-overexpressing cells were established by transfecting the cells with pBabepuro-EGFR plasmid and selected with puromycin at 1 µg/ml (designated as EGFR cells). Their corresponding controls were transfected with empty vector pBabepuro plasmid (designated as vector). Both plasmids were kindly provided by Prof. Paolo Di-Fiore, Department of Experimental Oncology, Istutito Europeo di Oncologia, Milan, Italy.

### Immunoblotting

Immunoblotting was performed following a previous study [33]. Briefly, 20 µg of cell lysates were separated using SDS-PAGE and proteins were transferred to a nitrocellulose membrane (GE Healthcare). The membrane was blocked with 5% non-fat milk and probed with a primary antibody at 4 °C overnight and then with the corresponding species of horseradish peroxidase-conjugated secondary antibody at 1:10,000 dilution. The blots were then visualised by chemiluminescence (LI-COR Odyssey Fc). The primary antibodies were diluted at 1:1000 for detection of immunoblotting except for the α-tubulin and β-actin antibodies which were diluted at 1:7000. Quantification of protein expression was achieved by measuring intensities of the protein of interest band adjusted for its loading control band (α-tubulin) then normalised using the control values. Band intensities were measured using ImageJ 1.53k.

### MTT assay for cell proliferation and viability

Cell survival was determined using 3-(4,5-dimethylthiazol-2-yl)-2,5-diphenyltetrazolium bromide (MTT) cell viability assay as previously described [34]. MTT is a commonly used cytotoxicity assay directly measures cellular metabolism and indirectly a measurement of cell viability. Optical density was measured using FlexStation 3 microplate reader (Molecular Devices) at 570 nm wavelength.

### Manipulation of miR-9-5p expression

Plasmids for overexpressing and knocking down hsa-miR-9-5p were previously established [28]. Scrambled sequences were used to construct the controls designated as miR-NC for overexpressing miR-9-5p, and KD-NC for knocking down of the miRNA. Cells were transfected with the plasmids for 24 hours using jetPRIME transfection reagent (Polyplus). After the transfection, cells were selected with G418 (500 ug/ml) for miR-9-5p overexpression and puromycin (1 µg/ml) for miR-9-5p knockdown. Surviving clones were allowed to expand and used for subsequent experiments.

### Quantitative RT-PCR

RNA isolation was done using TRIzol reagent (ThermoFisher) and qRT-PCR was performed using EVAGreen qPCR Mix (Thistle Scientific). The primers used for qRT-PCR were according to previous studies [35–38] as below. RNA expression was normalised using GADPH as housekeeping gene. Amplification was performed in Rotor-Gene 6000 real-time PCR machine (Corbett Life Science), and data were analysed using Rotor-Gene Q – Pure Detection Software (Qiagen).

BRD4-fw: 5′-AACCTGGCGTTTCCACGGTA-3′

BRD4-rev: 5′-GCCTGCACAGGAGGAGGATT-3′

HPV16 E6-fw: 5’-TCAGGACCCACAGGAGCG-3’

HPV16 E6-rev: 5’-CCTCACGTCGCAGTAACTGTTG-3’

HPV16 E7-fw: 5’-GAACCGGACAGAGCCCATTA-3’

HPV16 E7-rev: 5’-ACACTTGCAACAAAAGGTTACA-3’

GAPDH-fw: 5’-TGGATATTGTTGCCATCAATGACC-3’

GAPDH-Rev: 5’- GATGGCATGGACTGTGGTCAT-3’

Detection of miR-9-5p was previously described [28]. Hsa-miR-9-5p, RNU6B and RNU48 primers were from Applied Biosystems.

### Immunofluorescence

50,000 cells were seeded into 8-chamber slides (BD Biosciences). On the next day, cells were washed twice with 1× PBS and fixed with 4% paraformaldehyde solution for 20 minutes. Cells were then washed with 1× PBS thrice and were subjected to permeabilisation using 0.2% Triton-X100, then washed three times and incubated with 1% BSA in PBS for 60 minutes. Cells were incubated overnight at 4 °C with antibody of choice diluted in 1× PBS or simply 1× PBS as a negative control. Afterwards, cells were washed three times with 1× PBS and incubated with fluorescence-tagged secondary antibody for 90 minutes at room temperature protected from light. Following three times washing with 1× PBS, the chamber was removed from the slide and mounted with Vectashield mounting medium containing 4,6-diamidin-2-phenylindole (Vector Laboratories). Images were acquired using Zeiss LSM 700 microscope. The dilution used for BRD4 antibody was 1:200. Fluorescence intensities were measured using ImageJ 1.53k.

### P53 knockdown

pRetroSuper-blasto-p53i plasmid, a kind gift from Professor Dagmar Kulms, Department of Dermatology, Technical University Dresden, was transfected to generate p53 knockdown cells. The transfected cells were selected using cell culture medium containing 5 μg/ml blasticidin (ant-bl-1) from InvivoGen. P53 expression of each clone was determined using a western blot. selected clones with the lowest p53 expression level were used in further experiments.

### Database analysis

The Cancer Genome Atlas (TCGA) has been consulted to assess mRNA and protein levels in HNSCC patients [39]. GSE62944 and GSM1536837 datasets were used for correlation studies of gene expression levels [40], while the corresponding protein levels were investigated through TCGA RPPA dataset of HNSCC tumours. R CRAN ggplot2 and ggsignif libraries were used to plot and estimate the Pearson correlation coefficients and p-values [41, 42].

The top 20 downregulated genes for the EGF pathway in the human were extracted from PROGENy (PMID: 29295995) with the script available on Bioconductor (https://www.bioconductor.org/packages/release/bioc/html/progeny.html).

### Statistical analysis

All experiments were independently performed in triplicate. Data are expressed as mean ± SEM. Independent samples *t* test (two groups) and one-way analysis of variance (ANOVA) with Tukey’s post hoc test (three groups or more) were performed to compare the data. Two-way ANOVA with Tukey’s post hoc test was performed for the cell proliferation experiment. Kolmogorov–Smirnov test was used to test for normality. *P* < 0.05 was considered significant. Statistical analysis was performed using SPSS version 22.0.

### Reporting summary

Further information on research design is available in the Nature Research Reporting Summary linked to this article.

## Results

### Effects of EGFR overexpression on cell proliferation, p-ERK1/2 and E6/E7 expression in HPV-positive HNSCC

**Fig. 1:**
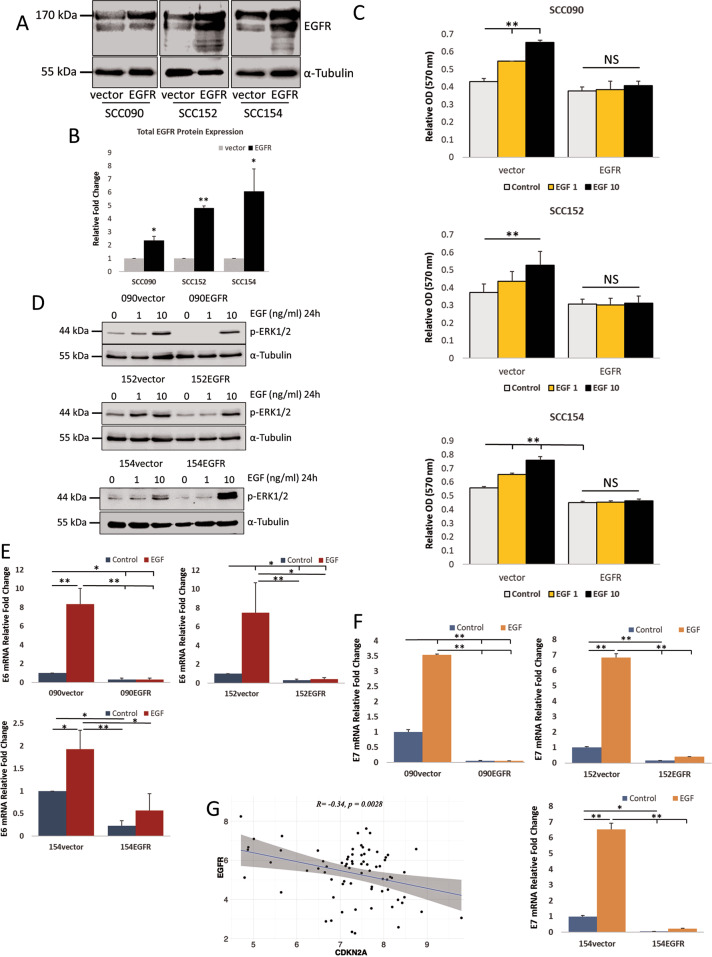
Effects of EGFR overexpression and EGF treatment in HNSCC cells. **A** EGFR expression in SCC090, SCC152 and SCC154 in EGFR-overexpressing cells and their control counterparts. **B** Quantification of three independent experiments of EGFR overexpression presented in bar-chart. Band intensities were adjusted for the intensities of the loading control bands then normalised using the control value, independent *t* test (*p < 0.05, **p < 0.01). **C** Cell proliferation measured by MTT at 72 h after EGF treatment (EGF 1 – 1 ng/ml; EGF 10 – 10 ng/ml) in vector control and EGFR-overexpressing cells. Statistical analysis was performed using two-way ANOVA with Tukey post hoc test (*p < 0.05, **p < 0.01). The full growth curve can be found in Supplementary Figure S1A. **D** Levels of phosphorylated ERK1/2 expression in EGFR-overexpressing SCC090, SCC152 and SCC154 cells and their control counterparts after EGF treatment at indicated concentrations for 24 h. HPV E6 (**E**) and HPV E7 (**F**) transcription after 10 ng/ml EGF treatment for 24 h measured by qRT-PCR in three independent RNA extractions. Statistical analysis was performed by one-way ANOVA (*p < 0.05, **p < 0.01). **G** Estimated Pearson correlation factor (R) for CDKN2A vs EGFR at mRNA levels in HPV-positive HNSCC samples from the TCGA database (GSE_62944_06_01_15_TCGA_24).

To investigate the roles of EGFR in HPV-related HNSCC, EGFR-overexpressing HPV-positive cell lines, SCC090 and SCC152, and their respective controls were generated in addition to SCC154 which was previously reported [12]. The expression of EGFR was significantly elevated in the polyclones of the established cells (Fig. 1A, B). In HPV-negative HNSCC, EGFR overexpression is known to promote tumour cell proliferation, disease progression and correlates with poor prognosis [43]. To determine the effect of EGFR overexpression in HPV-positive HNSCC, cell proliferation was measured by MTT assay with and without EGF treatment. EGFR-overexpressing cells were found to have lower proliferation rate than their controls (vector), EGF stimulation only enhanced cell proliferation in the vector control cells but not in the EGFR-overexpressing cells (Figs. 1C and S1A, B). EGFR is known to activate downstream targets through phosphorylation of ERK1/2 (p-ERK1/2) in HNSCC [31]. Therefore, the levels of p-ERK1/2 was determined by Western blot, EGFR-overexpressing cells showed less ERK1/2 activation at a lower concentration of EGF compared to the controls (Fig. 1D). Level of p-ERK1/2 has been linked with activation of HPV E6/E7 in cervical cancer cells [19], we then determined transcription of HPV E6 and E7 in the controls and EGFR-overexpressing HNSCC cell lines by qRT-PCR. EGFR overexpression significantly suppressed transcription of both HPV E6 and E7 in all three cell lines. Despite an increase in p-ERK1/2 level with EGF treatment, transcription of HPV E6 and E7 was not significantly increased in the EGFR-overexpressing cells treated with EGF at 10 ng/ml for 24 h, contrary to the control cells (Fig. 1E, F). These data suggest that EGFR overexpression decelerates HNSCC cell proliferation as well as transcription of HPV E6/E7 regardless of EGF stimulation. In support of our results that EGFR overexpression lowers the levels of HPV oncoproteins, the TCGA database analysis showed that expression of p16INK4a (or CDKN2A), an established surrogate marker for HPV, has a negative correlation with EGFR expression (Fig. 1G) [44]. In validation of this data, we found that EGFR overexpression suppressed p16INK4a expression in SCC154 cells (Fig. S1C). These results demonstrate that suppression of E6 and E7 transcription in HPV-associated HNSCC cell lines was EGFR-dependent.

### EGFR overexpression downregulates BRD4

**Fig. 2:**
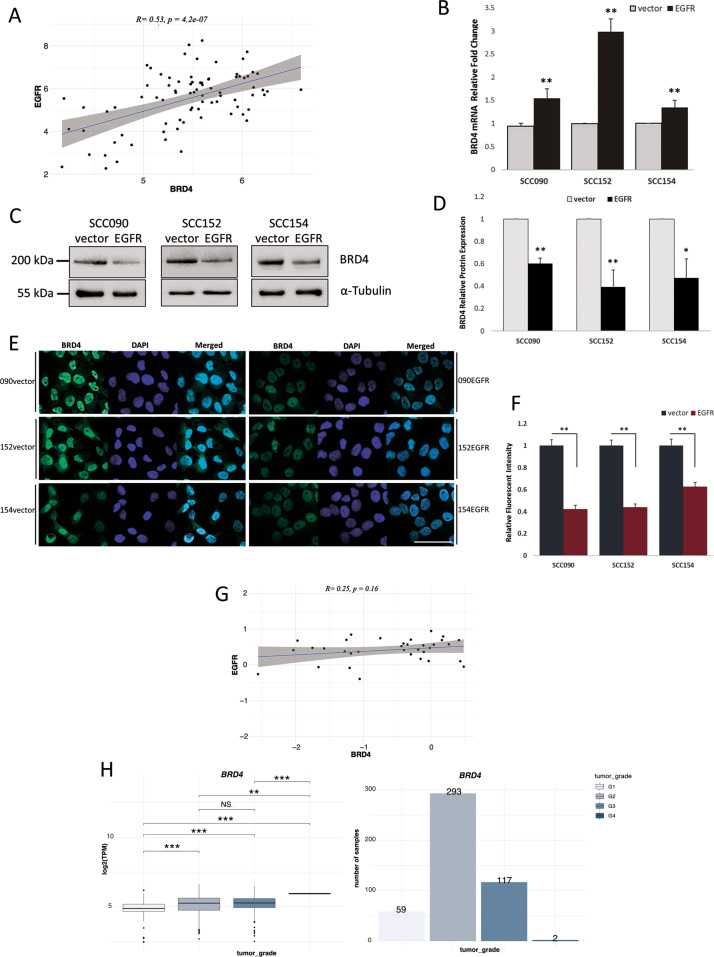
EGFR overexpression is associated with BRD4 in HPV-positive HNSCC cells. **A** Estimated Pearson correlation factor (R) at mRNA levels for BRD4 vs EGFR in HPV-positive HNSCC samples from the TCGA database. **B** BRD4 mRNA expression in SCC090, SCC152 and SCC154 cells and their EGFR-overexpressing counterparts as determined by qRT-PCR of three independent RNA extractions. Statistical analysis was performed by independent *t* test comparing each pair of the cells (vector vs EGFR) (**p < 0.01). **C** Protein expression of BRD4 determined by immunoblot and their quantification of three independent measurements of BRD4 expression presented in bar-chart (**D**), independent *t* test (*p < 0.05, **p < 0.01). **E** Fluorescence microscopy of BRD4 in HPV-positive cell line and their EGFR-overexpressing counterparts, scale bar–50 µm and quantification of the fluorescence intensity (**F**), independent *t* test (**p < 0.01). **G** Estimated Pearson correlation factor (R) at protein levels for BRD4 vs EGFR in HPV-positive samples from the TCGA database (TCGA RPPA). **H** BRD4 expression in different HNSCC tumour grades from the TCGA database, one-way ANOVA (**p < 0.01, ***p < 0.001, NS not significant) and abundances of samples for each category. Median and 25% and 75% quartiles are shown as box, 5% and 95% are visualised as whiskers. TPM values are log2 transformed.

An important protein involved in the transcription of HPV E6/E7 is BRD4, reported as a driver protein for HPV replication as well as HPV E6 expression [21]. Tandemly integrated HPV16 forms a BRD4-dependent superenhancer-like element and drives transcription of early viral oncogenes [23]. We analysed the TCGA database and found that EGFR and BRD4 mRNA expression levels were positively correlated in HPV-positive HNSCC (Fig. 2A) [44]. To further confirm the database results, qRT-PCR of BRD4 was performed in the three cell lines and the results were in agreement with the published database; overexpression of EGFR led to an increase in BRD4 mRNA (Fig. 2B). By contrast, immunoblotting clearly demonstrated that EGFR overexpression decreased BRD4 protein levels in all cell lines tested (Fig. 2C, D). Immunofluorescent staining of the cells detected BRD4 mostly concentrated in the cell nucleus and that EGFR overexpression reduced BRD4 protein expression (Fig. 2E, F). However, EGFR overexpression enhanced BRD4 expression in HPV-negative SCC072 cells (Fig. S1D, E). TCGA database analysis also suggested that, despite the significant correlation in their mRNA expression, EGFR and BRD4, at the protein levels, are no longer correlated in the HPV-positive group of patients (*p* = 0.16) (Fig. 2G) [44]. The TCGA database also identifies that grade 1 HNSCC tumour had significantly lower BRD4 expression than the higher grades (Fig. 2H) implicating that BRD4 is upregulated in higher grade tumours linking to their therapy resistance and that BRD4 could be a therapeutic target in HPV-positive HNSCC.

### Effects of activation and inhibition of EGFR on BRD4

**Fig. 3:**
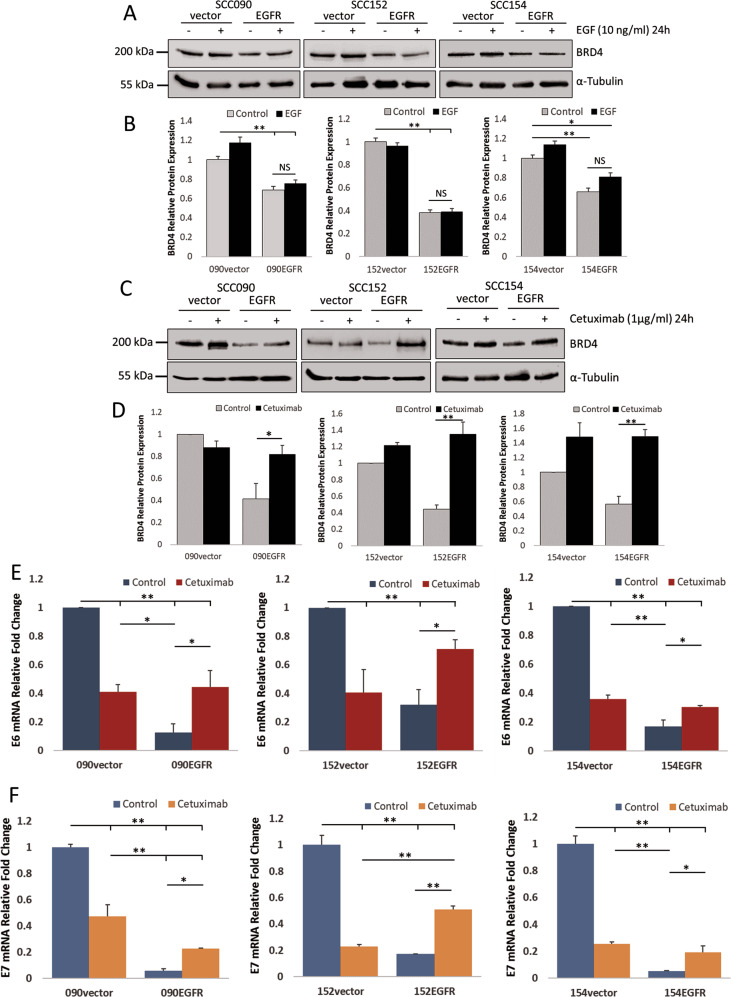
Responses of HPV-positive HNSCC cells after EGF and cetuximab treatments. **A** Expression of BRD4 after 24 h of EGF treatment (10 ng/ml). **B** Quantification of three independent measurements of BRD4 protein expression after the EGF treatment presented in bar-chart. Statistical analysis was performed by one-way ANOVA (*p < 0.05, **p < 0.01, NS not significant). **C** Expression of BRD4 after treatments with cetuximab (1 µg/ml 24 h). **D** Quantification of three independent measurements of BRD4 protein expression after the cetuximab treatment presented in bar-chart. Statistical analysis was performed by one-way ANOVA (*p < 0.05, **p < 0.01); only comparisons for EGFR-overexpressing cells are shown. HPV E6 (**E**) and HPV E7 (**F**) transcription after cetuximab treatment (1 µg/ml 24 h) measured by qRT-PCR in three independent RNA extractions. Statistical analysis was performed by one-way ANOVA (*p < 0.05, **p < 0.01).

To investigate EGFR-dependent regulation of BRD4, cells were treated with recombinant EGF. Activation of EGFR with EGF treatment failed to induce BRD4 expression in the HPV-positive HNSCC cells (Fig. 3A, B). Furthermore, inhibition of EGFR with cetuximab (1 µg/ml for 24 h) demonstrated that cetuximab had little effect on BRD4 expression in the control cells (Fig. 3C, D). However, BRD4 expression was significantly enhanced by cetuximab treatment in EGFR-overexpressing cells (Fig. 3C, D). The effect of cetuximab on HPV E6/E7 transcription was then determined. Cetuximab treatment lowered HPV E6 and E7 in the control cells, while restoring HPV E6 and E7 levels in the EGFR-overexpressing cells confirming EGFR-dependent suppression of these viral oncoproteins (Fig. 3E, F). The restoration of BRD4 expression by cetuximab could potentially be the cause of HPV E6/E7 resurgence in the EGFR-overexpressing cells. Meanwhile, cetuximab treatment in HPV-negative SCC072 had trivial effect on BRD4 expression (Fig. S1F). The same treatment also failed to induce levels of p-ERK1/2 (Fig. S1G) making BRD4 the likely candidate that caused the restoration of HPV E6/E7 transcription. We also attempted to verify whether BRD4 is linked to MAPK pathway which may cause an increase in HPV E6/E7 levels. Using a MEK1/2 inhibitor selumetinib, the treatment with selumetinib (1 µM, 24 h) had minimal effect on BRD4 expression in all cell lines (Fig. S1H). These results, together with those in Fig. 1D–F, suggest that EGFR in HPV-positive HNSCC regulates BRD4 and HPV oncoprotein expression in a MAPK-independent manner.

### miR-9-5p regulates expression of BRD4 and subsequent HPV E6/E7

**Fig. 4:**
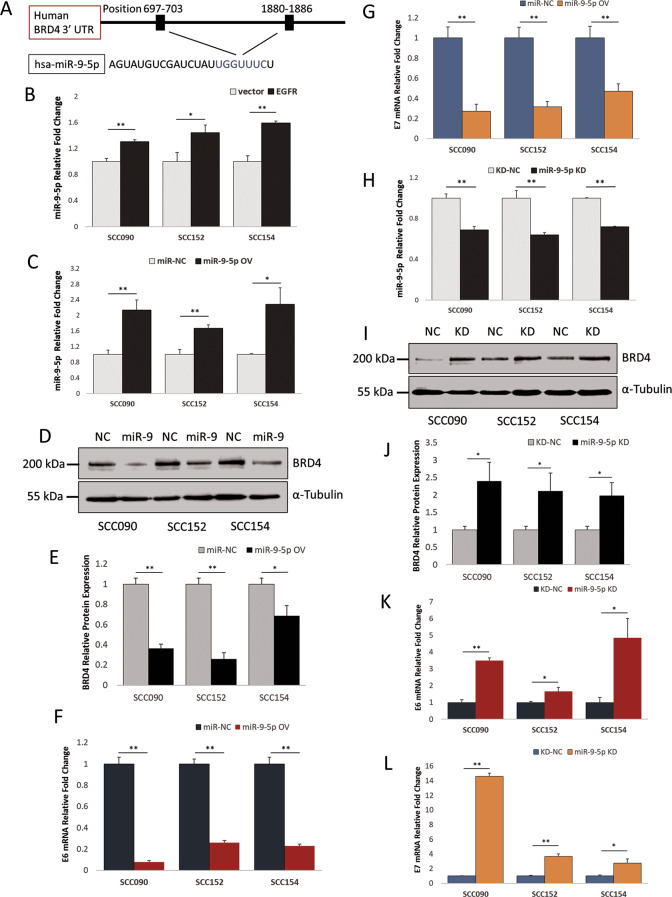
miR-9-5p modulates BRD4 and HPV E6/E7 expression. **A** Binding sites of miR-9-5p in the 3’UTR of human BRD4 predicted by TargetScan. **B** miR-9-5p expression in SCC090, SCC152 and SCC154 control cells and their EGFR-overexpressing counterparts as determined by qRT-PCR of three independent RNA extractions. Statistical analysis was performed by independent *t* test comparing each pair of the cells (vector vs EGFR) (*p < 0.05, **p < 0.01). **C** miR-9-5p expression in miR-9-5p overexpressing cells (miR-9-5p OV) as determined by qRT-PCR of three independent RNA extractions, independent *t* test (*p < 0.05, **p < 0.01), miR-NC – control (scrambled sequence). **D** BRD4 expression in SCC090, SCC152 and SCC154 in miR-9-5p (miR-9) overexpressing cells and their control counterparts (NC). **E** Quantification of three independent measurements of BRD4 expression in miR-9-5p overexpressing cells presented in bar-chart, independent *t* test (*p < 0.05, **p < 0.01). HPV E6 (**F**) and HPV E7 (**G**) transcription in miR-9-5p overexpressing cells, comparisons were made within each cell line using independent *t* test (**p < 0.01). **H** miR-9-5p expression in miR-9-5p knockdown cells (miR-9-5p KD) as determined by qRT-PCR of three independent RNA extractions, independent *t* test (**p < 0.01), KD-NC – control (scrambled sequence). **I** BRD4 expression in SCC090, SCC152 and SCC154 in miR-9-5p (miR-9) knockdown cells and their control counterparts (NC) and their quantification of three independent measurements of BRD4 expression in miR-9-5p knockdown cells presented in bar-chart (**J**), independent *t* test (*p < 0.05), (**K**) and HPV E7 (**L**) transcription in miR-9-5p overexpressing cells, comparisons were made within each cell line using independent *t* test (*p < 0.05, **p < 0.01).

To determine the mechanism by which EGFR suppresses BRD4 protein expression, we looked for potential microRNA candidates using TargetScan and found that human miR-9-5p is predicted to have two possible binding sites to 3’UTR of BRD4, as shown in Fig. 4A. We then measured the expression of miR-9-5p and found that the EGFR-overexpressing HPV-positive HNSCC cells had higher levels of miR-9-5p than their control counterparts (Fig. 4B). EGFR overexpression also elevated the level of miR-9-5p in HPV-negative SCC072 cells, but this was not statistically significant (Fig. S2A). To further test the importance of miR-9-5p in HPV-positive HNSCC, we established miR-9-5p overexpressing cell lines and confirmed the overexpression (Figs. 4C, S2B). Protein levels of BRD4 were then determined and found to be significantly less in miR-9-5p overexpressing cells (Fig. 4D, E). MiR-9-5p overexpression also lowered BRD4 protein expression in SCC072 (Fig. S2C, D). Next, transcription levels of HPV E6/E7 were determined, and overexpression of miR-9-5p significantly reduced the levels of both E6 and E7 (Fig. 4F, G). To further verify the results, miR-9-5p knockdowns of the cell lines were established and the levels of the microRNA were confirmed (Figs. 4H and S2E). Expectedly, BRD4 protein expression was enhanced by the knockdown for both the HPV-positive cell lines (Fig. 4I, J) and the HPV-negative cell line (Fig. S2F, G). The miR-9-5p knockdown subsequently induced the expression of HPV E6/E7 (Fig. 4K, L). These data suggest that EGFR overexpression enhances miR-9-5p level which then negatively regulates BRD4 protein translation causing a reduction in HPV E6/E7 transcription.

### Inhibition of BRD4 suppresses E6/E7 transcription and radiosensitises HNSCC cells

**Fig. 5:**
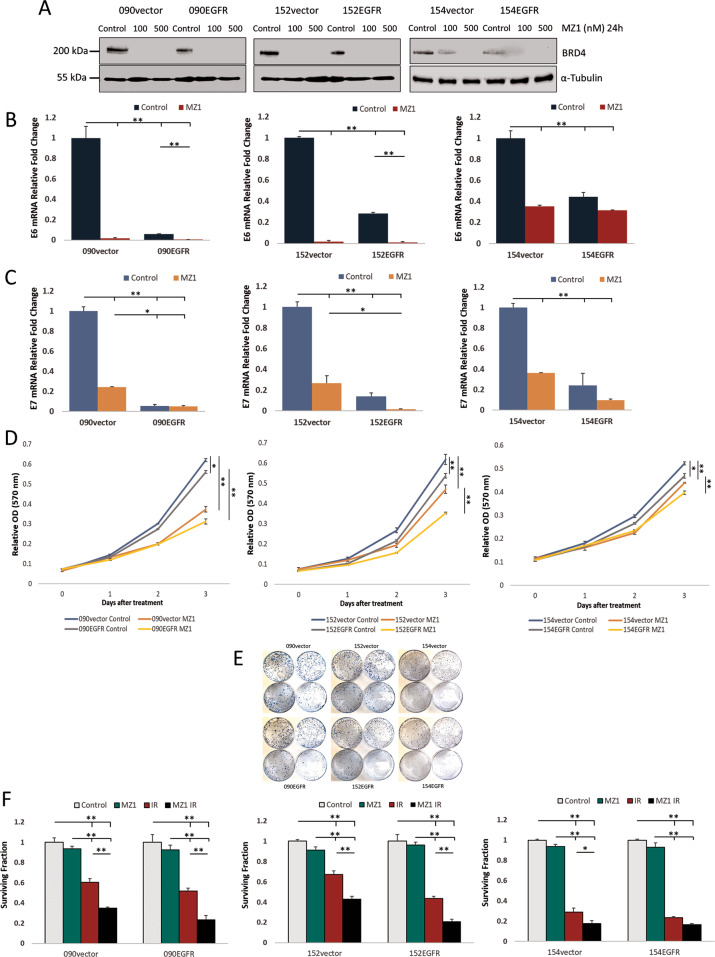
Effects of BRD4 inhibition using MZ1 inhibitor. **A** Expression levels of BRD4 in SCC090, SCC152 and SCC154 control cells and their EGFR-overexpressing counterparts as determined by western blot after MZ1 treatment at indicated concentrations for 24 h. **B** HPV E6 and **C** HPV E7 transcription after MZ1 treatment (100 nM for 24 h) determined by qRT-PCR in three independent RNA extractions. Statistical analysis was performed by one-way ANOVA (*p < 0.05, **p < 0.01). **D** Cell proliferation measured by MTT from day 0-3 treated with MZ1 (100 nM). Statistical analysis was performed using two-way ANOVA (*p < 0.05, **p < 0.01). **E** Clonogenicity after 5 Gy irradiation of the HPV-positive control cells and their EGFR-overexpressing counterparts treated with MZ1 prior to irradiation (in each panel, top left- untreated control; top right- MZ1 (100 nM 24 h); bottom left- 5 Gy irradiated; bottom right- MZ1 (100 nM 24 h) with 5 Gy irradiation). **F** Quantification of clonogenic survival treated with the same conditions in three independent experiments. Control untreated control, MZ1 MZ1 100 nM 24 h; IR- 5 Gy irradiated; MZ1 IR – MZ1 100 nM 24 h then 5 Gy irradiated. Statistical analysis was performed using one-way ANOVA (*p < 0.05, **p < 0.01).

To further explore the importance of BRD4 in HPV-positive HNSCC, a BRD4 inhibitor, MZ1, was used; this inhibitor is a proteolysis targeting chimera (PROTAC) which causes degradation of the target protein [45]. We selected this inhibitor because the EGFR-overexpressing cells had lower expression of BRD4; to observe the effect of lowered BRD4 expression, a degrading inhibitor was preferred over non-degrading inhibitor such as JQ1 which is commonly used in various BRD4-related studies. As shown in Fig. 5A, treatment with as low as 100 nM concentration of MZ1, clearly suppressed BRD4 protein levels. The effect of BRD4 inhibition on HPV E6/E7 transcription was verified by qRT-PCR. The results show that MZ1 treatment significantly lowered HPV E6 and E7 transcription in all control cells (Fig. 5B, C). As EGFR-overexpressing cells already had relatively low levels of HPV E6/E7 transcription, MZ1 treatment had limited effect on lowering their transcription. To determine the effect of MZ1 on cell proliferation, MTT assay demonstrated that MZ1 treatment significantly decreased HNSCC cell proliferation (Figs. 5D and S3A). Furthermore, MZ1 was used to treat the cells prior to irradiation to determine its ability to radiosensitise the HNSCC cells. At the concentration used (100 nM), MZ1 was not toxic to the cells (Fig. S3B). BRD4 inhibition appeared to increase radiosensitivity and reduce survival measured by clonogenicity (Fig. 5E, F). This is also the case for HPV-negative SCC072 as shown in Fig. S3C, D. Additionally, BRD4 has been shown to associate with DNA damage repair with inhibition of BRD4 causing reduction in expression of DNA damage repair-related genes including RAD51AP1 and CDC6 [46, 47]. Our analysis of the TCGA database in HPV-positive HNSCC shows that, at mRNA levels, BRD4 expression was positively correlated with RAD51AP1, CDC6 as well as EGFR. However, EGFR expression had no significance correlations with RAD51AP1 or CDC6 (Fig. S4A). Likewise, we looked into HPV-positive HNSCC tumours with different grades in the TCGA and found that higher grade tumours appeared to have higher expression of BRD4, CDKNA2, RAD51AP1 and CDC6 with EGFR expression was lesser in higher tumour grades (Fig. S4B) [44]. To further verify that BRD4 expression is not only correlated with EGFR expression, but also with its estimated activity, we used PROGENy, which gives a signature of the pathway responsiveness, to select the top 20 downregulated EGFR target genes and inferred their correlation with BRD4 as a proxy to the pathway activity. Figure S5 showing Pearson correlation score for each pair of the targets, reveals that there is a relatively strong signal coming from EGFR as all the selected genes have positive correlations. Similarly, they are positively correlated to BRD4; thus, the data supports that BRD4 is downregulated under the EGFR pathway activity in HPV-positive samples. These data further implicate the importance of alternative EGFR roles and its implication for therapy response in HPV-positive HNSCC through a potential role in suppression of BRD4-related genes.

### EGFR-induced radiosensitisation in HPV-positive HNSCC is p53 dependent

**Fig. 6:**
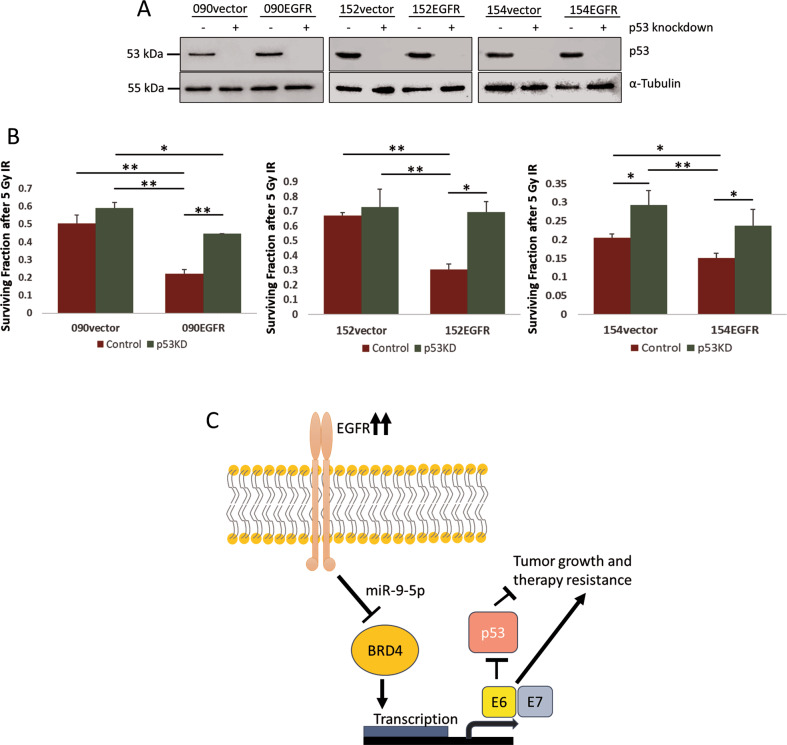
Knockdown of p53 reverts EGFR-mediated radiosensitisation. **A** Levels of p53 in the selected clones of SCC090, SCC152 and SCC154 with minimal p53 expression in the controls and EGFR-overexpressing cells after transfection with pRetroSuper-blasto-p53i plasmid (p53 shRNA). **B** Clonogenic survival of p53 knocked down (p53KD) cells after 5 Gy irradiation (IR) in four independent experiments. Statistical analysis was performed by one-way ANOVA with Tukey post hoc test (*p < 0.05, **p < 0.01). **C** Schematic representation of the mechanism involved in EGFR-associated transcription of HPV E6/E7 involving BRD4 and miR-9-5p.

We previously reported that EGFR overexpression could impair DNA damage repair and induce radiosensitivity in HPV-positive HNSCC cells [12]. Here, we further investigated whether radio-sensitization was due to p53 reactivation, as p53 remains intact in most of the HPV-related HNSCC. According to the TCGA database (PanCancer Atlas) through cBioPortal, 9 out of 72 cases of HPV-positive HNSCC contained mutations in p53 gene [48, 49]. To determine the role of p53, we knocked it down using shRNA. Successful p53 knockdown in selected clones was determined by western blotting (Fig. 6A). The cells were then irradiated and assessed by clonogenicity; p53 knockdown in EGFR-overexpressing cells led to a significant enhancement in radioresistance (Fig. 6B). Figure S6A indicates that p73, but not p63, expression was affected by p53 knockdown, and that EGFR overexpression induced p73 expression which could contribute to the improved radiosensitivity in HNSCC cells [50]. Additionally, the role of p53 in HPV E6 transcription, as HPV E6 binds to p53 to induces p53 protein degradation [51], was determined by qRT-PCR. Figure S6B shows that the knockdown had no obvious effect on HPV E6 transcription suggesting p53 may play limited role in regulating transcription of this viral oncoprotein.

## Discussion

The incidence of HPV-positive HNSCC is rising generally affecting younger individuals. It has become clear that HPV-driven HNSCC have a better treatment outcome; however, there are currently no subtype-specific treatments available and all HNSCC are treated with highly toxic standard RT/CT causing severe morbidities. Several studies have tested the effect of the only available targeted therapy, cetuximab, for the purpose of treatment de-escalation in HPV-driven cancers. Although targeting EGFR using cetuximab has shown some benefits for HPV-negative tumours, the outcome of EGFR inhibition for HPV-positive HNSCC was the opposite causing a clear inferior response and poorer survival outcome as compared with standard treatment modalities. We recently reported opposing roles for EGFR signalling between HPV-negative and positive HNSCC, where in the latter EGFR was a positive prognostic marker, delaying tumour cell proliferation and inhibiting DNA damage repair leading to radiosensitivity. Furthermore, we demonstrated EGFR overexpression lead to E6 suppression restoring p53 activity [12]. In this study, we sought to gain insight into the mechanism by which EGFR regulates HPV oncoproteins E6 and E7 causing radiosensitivity.

We demonstrated that EGFR overexpression downregulated both E6 and E7, which could be restored by cetuximab treatment suggesting an EGFR-dependent transcriptional regulation of these viral oncoproteins. Several cellular transcriptional regulators of HPV early genes have been reported including ERK1/2, p38, SOX2, Tip60 and p300 [52–54]. Additionally, protein BRD4 plays an important role in HPV viral DNA replication by binding to HPV E2 to stabilise HPV E2 association with chromatin [24]. HPV E2 then binds to the viral promoter next to the origin of replication and regulates the expression of HPV E6/E7 [55]. We, therefore, investigated whether EGFR overexpression affects BRD4 expression. Both the TCGA database analysis and qRT-PCR of the cell lines discovered a positive correlation between EGFR and BRD4 at mRNA expression levels. However, the reverse was observed with protein expression analyses where EGFR overexpression significantly reduced BRD4 protein levels, suggesting the potential epigenetic regulatory mechanisms, such as microRNAs that may translationally regulate BRD4. MicroRNA-9-5p is suggested to be a tumour suppressor in HNSCC and has been shown to have a number of potential favourable roles for HNSCC patients, particularly those with HPV-related subtype [28–30, 56]. The microRNA is often significantly upregulated in HPV-positive oropharyngeal squamous cell carcinoma [57]. Herein, we demonstrated the inhibition of BRD4 and consequently E6/E7 suppression by miR-9-5p which further strengthens its roles as a tumour suppressor in HPV-positive HNSCC. Its inhibitory effect on BRD4 protein expression complements our previous findings showing that EGFR overexpression leads to impairment of DNA damage repair in HPV-positive HNSCC as BRD4 is known to promote DNA repair [12, 23]. Besides the negative regulation by miR-9-5p, BRD4 can potentially be moderated by ubiquitination and degradation via Nedd4, activation of the ubiquitin ligase Nedd4 can be mediated by EGFR through PI3K pathway [58, 59]. Further mechanisms in the post-translational regulation of BRD4 remain to be investigated.

BRD4 is known as a part of super-enhancers in addition to being an epigenetic and transcriptional regulator. Inhibition of BRD4 limits the communication between super-enhancers and promoters for oncogene transcription [23] and inhibition of BRD4 has shown to downregulate Snail affecting EMT and metastatic potential of breast and gastric cancers [60, 61]. The protein also has a potential role in DNA damage repair by enhancing chromatin insulation to modulate signalling for DNA damage response [46, 62]. Therefore, targeting BRD4 could potentially benefit patients particularly when EGFR inhibition has failed to improve therapeutic outcome. Additionally, BRD4 inhibition could overcome cetuximab resistance in HNSCC highlighting that targeting EGFR using cetuximab would activate BRD4-dependent activation of other receptor tyrosine kinases including HER3, MET, and AXL [63]. There is a number of BRD4 inhibitors available with some in clinical trials such as AZD5153 in relapsed/refractory (R/R) solid tumours and lymphoma, CPI0610 in R/R lymphoma and myelofibrosis, and INCB057643 in several tumour types [64].

The link between EGFR/MAPK/ERK signalling cascade and its downstream activation of tumour cell proliferation has been well-established [19, 65]. The current study identifies a MAPK-independent pathway by which EGFR regulates HPV viral gene transcription. As proposed in the model in Fig. 6C, in the presence of viral genes, EGFR could adopt an alternative pathway through miR-9-5p resulting in the suppression BRD4 as well as HPV E6/E7 oncoproteins in HPV-positive HNSCC consequently improving treatment response. As this effect was mediated through BRD4, inhibition of the protein can provide a potential treatment option to enhance radiosensitivity of HPV-positive HNSCC. Our results are also in accordance with previous studies presenting that HPV-positive HNSCC with p16 expression tended to have low EGFR expression [66, 67]. Recent advances have been introduced to specifically target HPV oncoproteins including introduction of CRISPR/Cas9 and delivery of siRNA via Lipid-based nanoparticles hoping for an improvement in therapeutic outcomes of HPV-related cancers [68, 69]. Further studies should expand to establish a role for EGFR/BRD4/miR-9-5p axis in tumour itself and tumour microenvironment towards the development of better, less toxic and more efficient therapeutics for HPV-infected cancers [70].

In summary, the current study elucidates the connection between EGFR and BRD4 through miR-9-5p in HPV-positive HNSCC, which subsequently governs the transcription of HPV oncoproteins E6 and E7. Inhibition of BRD4 could lead to sensitisation of HNSCC to ionising radiation and potentially improve therapeutic outcomes in the patients.

## Supplementary information

Figure S1

Figure S2

Figure S3

Figure S4

Figure S5

Figure S6

Supplementary Figure Legends

Uncropped western blot images

Reporting Summary

## Data Availability

The data used to support the findings of the study are available from the corresponding author upon reasonable request.
