# Supplementary figures and images for: EGFR-induced suppression of HPV E6/E7 is mediated by microRNA-9-5p silencing of BRD4 protein in HPV-positive head and neck squamous cell carcinoma
Source: Cell Death Dis. 2022 Nov 4;13(11):921. doi: 10.1038/s41419-022-05269-8 (PMC9636399; doi:10.1038/s41419-022-05269-8)

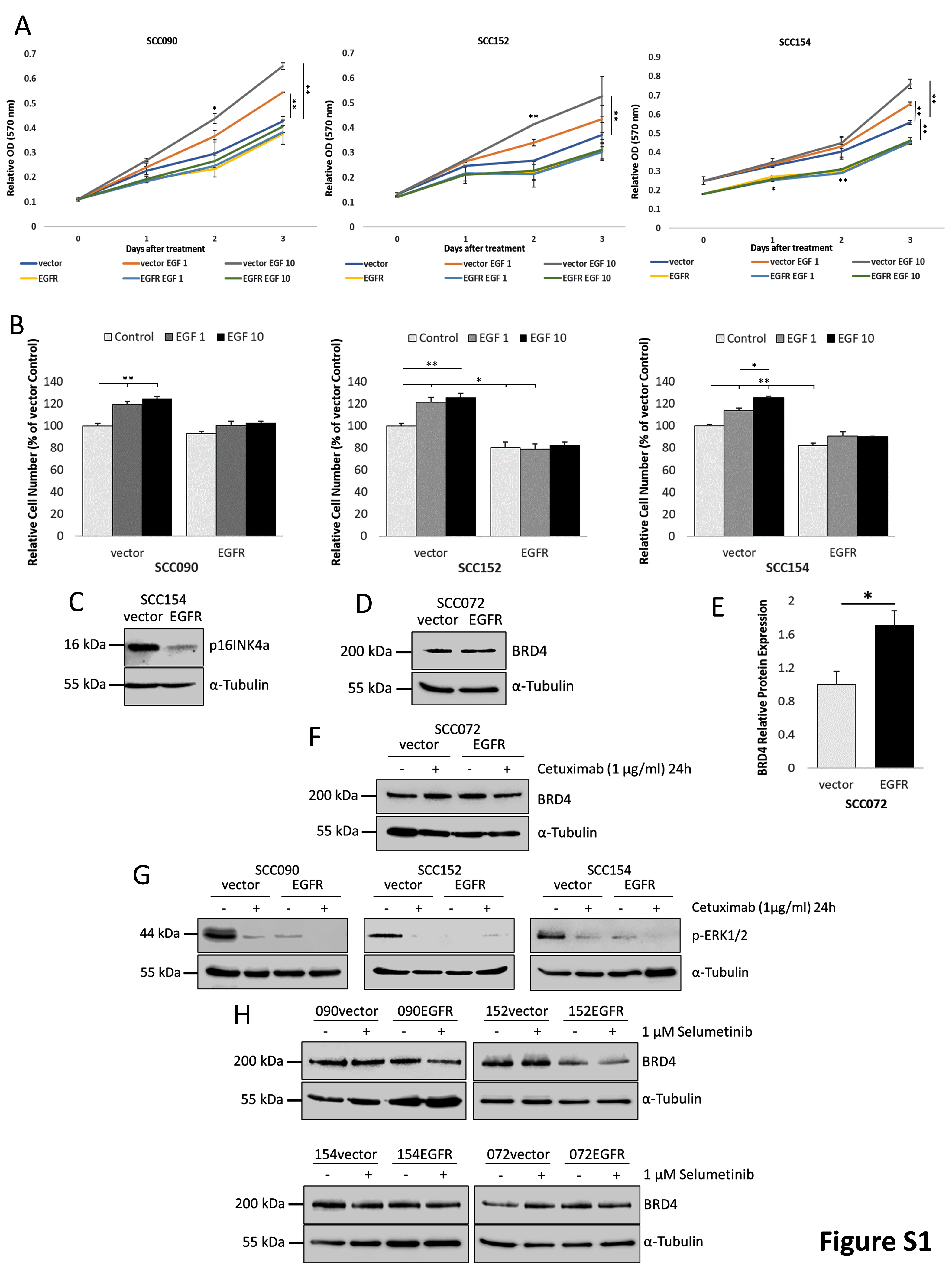

Supplement: Supplementary file 1 — Figure S1 [file 41419_2022_5269_MOESM1_ESM.tif]

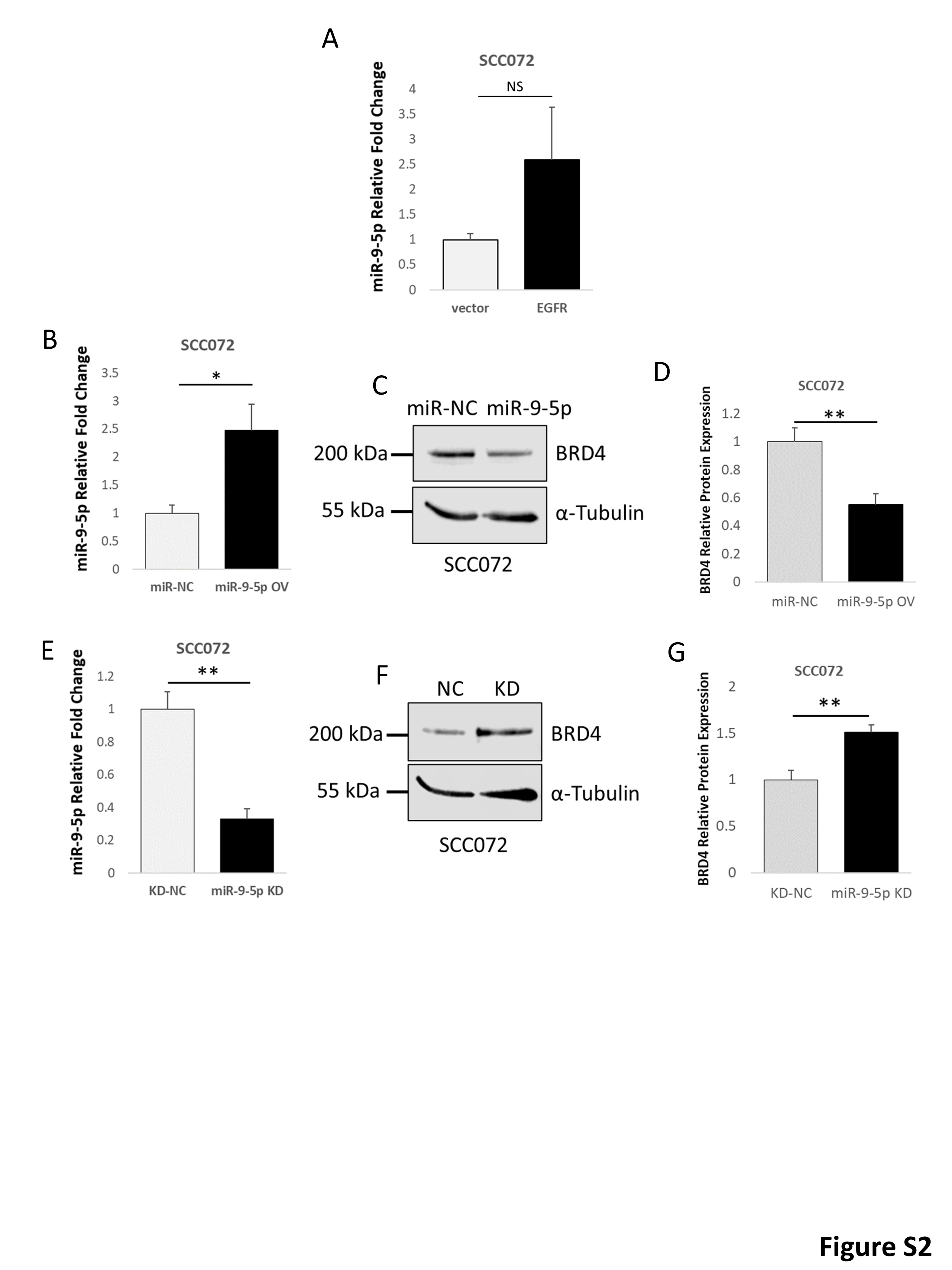

Supplement: Supplementary file 2 — Figure S2 [file 41419_2022_5269_MOESM2_ESM.tif]

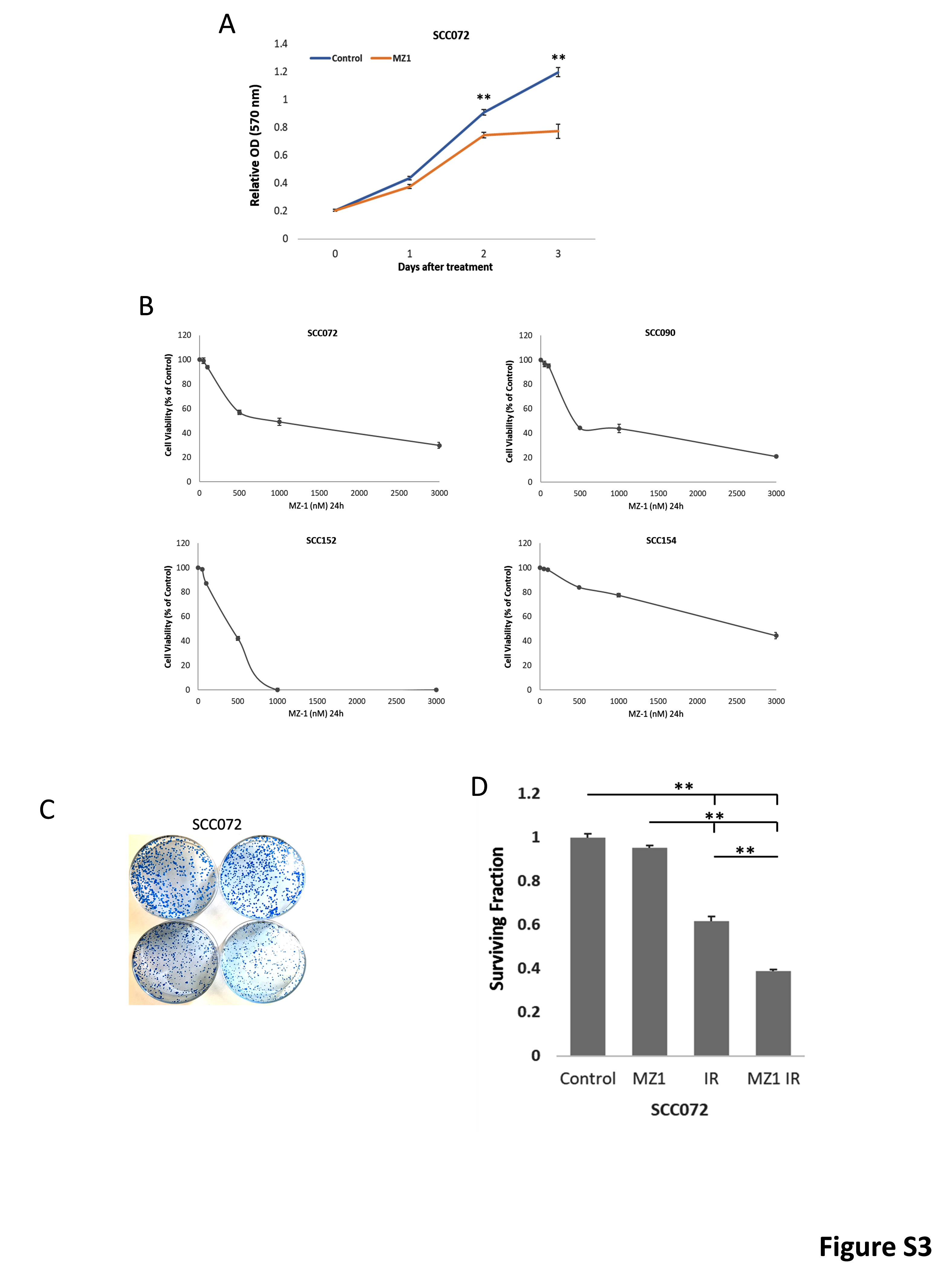

Supplement: Supplementary file 3 — Figure S3 [file 41419_2022_5269_MOESM3_ESM.tif]

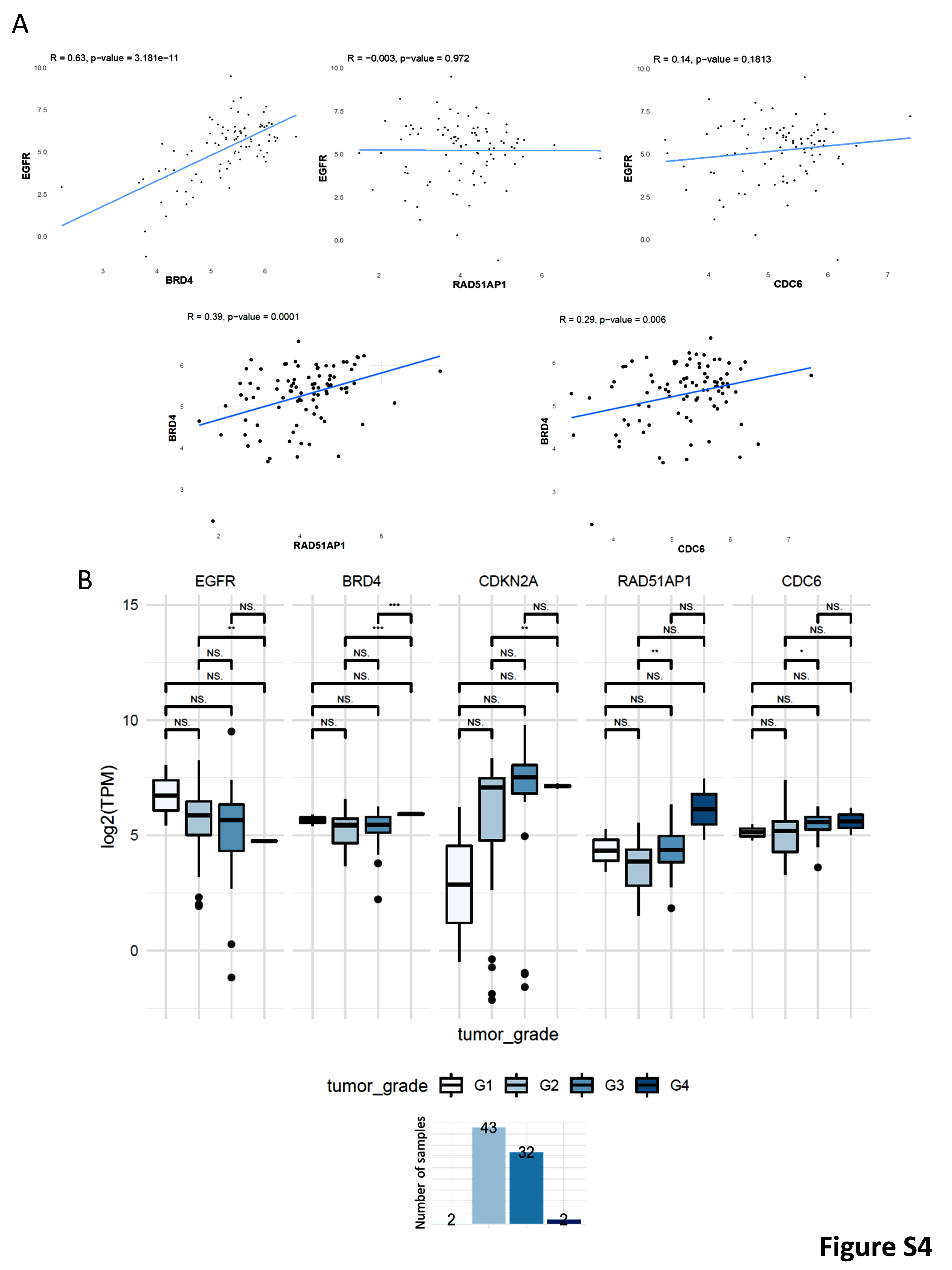

Supplement: Supplementary file 4 — Figure S4 [file 41419_2022_5269_MOESM4_ESM.tif]

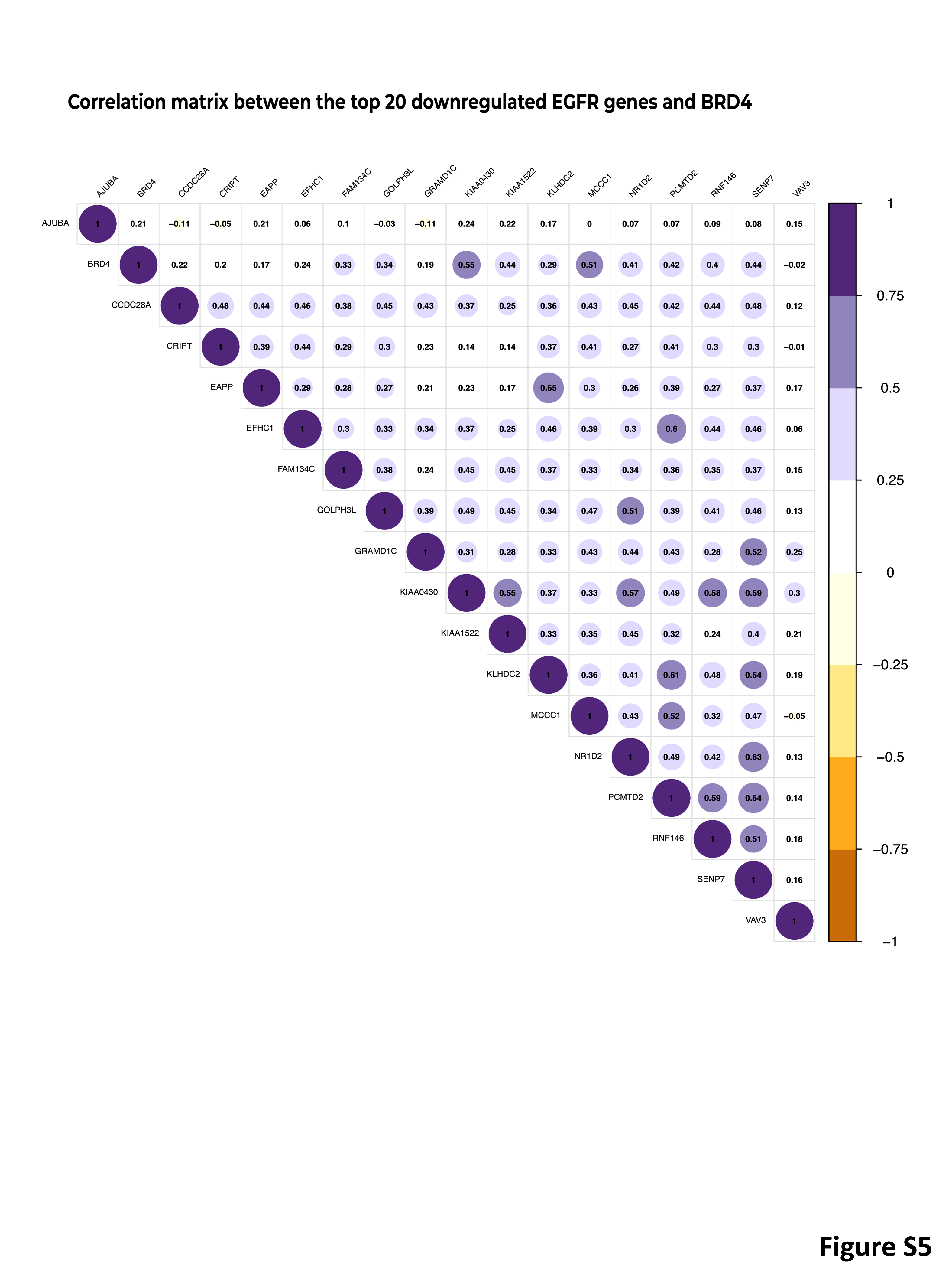

Supplement: Supplementary file 5 — Figure S5 [file 41419_2022_5269_MOESM5_ESM.tif]

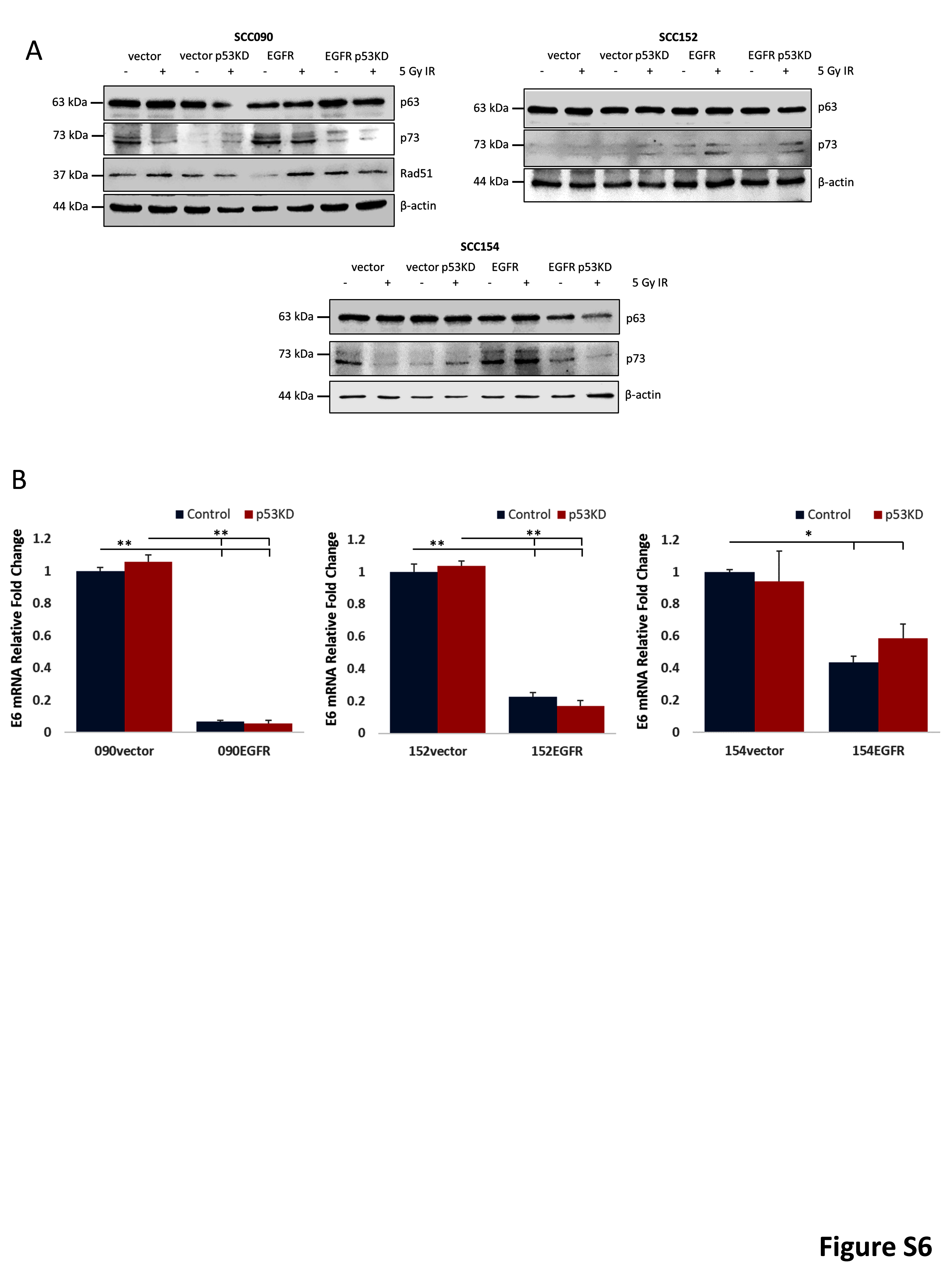

Supplement: Supplementary file 6 — Figure S6 [file 41419_2022_5269_MOESM6_ESM.tif]

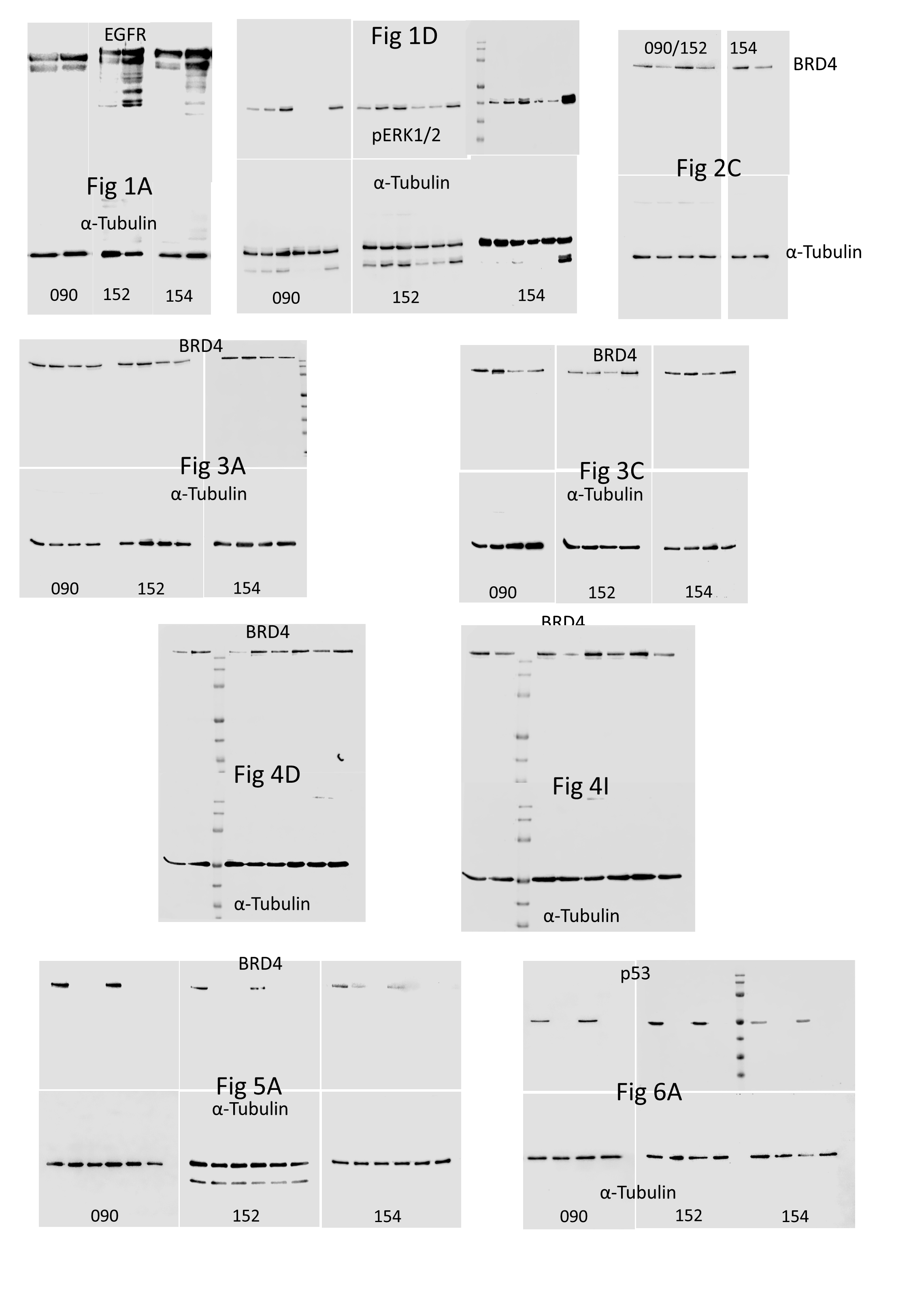

Supplement: Supplementary file 8 — Uncropped western blot images [file 41419_2022_5269_MOESM8_ESM.tif]
