# Supplementary material for: EGFR-induced suppression of HPV E6/E7 is mediated by microRNA-9-5p silencing of BRD4 protein in HPV-positive head and neck squamous cell carcinoma
Source: Cell Death Dis. 2022 Nov 4;13(11):921. doi: 10.1038/s41419-022-05269-8 (PMC9636399; doi:10.1038/s41419-022-05269-8)
Supplement: Supplementary file 7 — Supplementary Figure Legends [file 41419_2022_5269_MOESM7_ESM.docx]

**Supplementary figure legends**

**Fig. S1.** Expression of different proteins in HNSCC cells. (A) Three independent experiments of cell proliferation as determined by MTT assay from day 0-3 treated with 1 (EGF 1) and 10 (EGF 10) ng/ml of EGF concentrations. Statistical analysis was performed using two-way ANOVA with Tukey post-hoc test (*p<0.05, **p < 0.01). (B) Cell counts at 72 h after EGF treatment with 0 (control), 1 (EGF 1) and 10 (EGF 10) ng/ml in the complete culture medium. Statistical analysis was performed by one-way ANOVA with Tukey post-hoc test (*p<0.05, **p < 0.01); only comparisons with vector control are shown. (C) Expression of p16INK4a in SCC154 control and EGFR overexpressing cells. (D) BRD4 expression in SCC072 control and EGFR overexpressing cells along with their densitometric quantification adjusted with their loading control band intensities (E), independent t test (*p<0.05). (F) BRD4 expression in SCC072 after cetuximab treatment (1 µg/ml 24 h). (G) Expression of phosphorylated ERK1/2 in SCC090, SCC152 and SCC154 control and EGFR overexpressing cells treated with cetuximab (1 µg/ml 24 h). (H) Expression of BRD4 in the controls and EGFR overexpressing cells treated with 1 µM selumetinib for 24 h.

**Fig. S2.** miR-9-5p expression and effect in SCC072 cells. (A) Relative miR-9-5p expression in SCC072 control and EGFR overexpressing cells as determined qPCR from 3 independent extractions, independent t test (NS – not significant). (B) miR-9-5p expression in miR-9-5p overexpressing SCC072 cells (miR-9-5p OV) as determined by qRT-PCR of 3 independent RNA extractions, independent t-test (*p<0.05), miR-NC – control. (C) BRD4 expression in SCC072 miR-9-5p (miR-9-5p) overexpressing cells and its control counterpart and their densitometric quantification (D) (**p < 0.01). (E) miR-9-5p expression in miR-9-5p knockdown SCC072 cells (miR-9-5p KD) as determined by qRT-PCR of 3 independent RNA extractions, independent t-test (*p<0.05, **p < 0.01), KD-NC – control. (F) BRD4 expression in SCC072 miR-9-5p (miR-9) knockdown cells and its control counterpart and their densitometric quantification (G) (**p < 0.01).

**Fig. S3.** Effects of MZ1 treatment in HNSCC cells. (A) Cell proliferation determined by MTT in SCC072 cells after treatment with BRD4 inhibitor, MZ1, at 100nM. (B) Cell viability of different HNSCC cells determined by MTT after 24 hours treatment of MZ1 at indicated concentrations. (C) Clonogenicity after MZ1 treatment with 5 Gy irradiation in SCC072 cells (in the panel, top left- untreated control; top right- MZ1 (100nM 24h); bottom left- 5 Gy irradiated; bottom right- MZ1 (100nM 24h) with 5 Gy irradiation;). (D) Quantification of colonogenic survival in SCC072 cells treated with the same conditions in 3 independent experiments. Control – untreated control; MZ1 – MZ1 100 nM 24h; IR- 5 Gy irradiated; MZ1 IR – MZ1 100nM 24h then 5 Gy irradiated. Statistical analysis was performed by one-way ANOVA with Tukey post-hoc test (*p<0.05, **p < 0.01).

**Fig S4.** TCGA analysis of EGFR, BRD4 and DNA repair associated genes in HPV-positive HNSCC. (A) Estimated Pearson correlation factor (R) for designated pairs of genes at mRNA levels in HPV-positive HNSCC samples from the TCGA database. (B) Indicated genes expression in different HNSCC tumour grades from the TCGA database, one-way ANOVA (*p < 0.05, **p < 0.01, NS - not significant) and the abundances of samples. Median and 25% and 75% quartiles are shown as box, 5% and 95% are visualized as whiskers. TPM values are log2 transformed. The datasets used were GSM1536837_06_01_15_TCGA_24 for the correlations, and GSE62944_06_01_15_TCGA_24_548_Clinical_Variables for the tumour grade analysis.

**Fig. S5.** Correlations between pairs of the top 20 EGFR downregulated genes along with BRD4 correlation with each gene within the cluster. The correlation coefficients vary from -1 to 0 for negative correlations, and from 0 to 1 for positive correlation. Colour and size of the bubbles stand for the strength of the correlations which are also expressed by the Pearson correlation score in the lines.

**Fig. S6.** Knocking down of p53 in HPV-positive cell lines. (A) Expression of p53, p63 and p73 in the 53 knocked down cells; also, Rad51 expression is included for SCC090 cells. (B) HPV E6 transcription in the selected clones of p53 knockdown cells. Statistical analysis was performed by one-way ANOVA with Tukey post-hoc test (*p<0.05, **p < 0.01).
